# Assessment of awareness and knowledge regarding nuclear medicine and appropriate use of Nuclear medicine modalities, among medical students and faculty members in two academic medical institutes in North India: A Cross sectional Study

**DOI:** 10.22038/AOJNMB.2023.71375.1497

**Published:** 2024

**Authors:** Nitin Gupta, Priya Sareen, Sudesh Kumar, Muninder Negi

**Affiliations:** 1 Department of Nuclear medicine, Dr Rajendra Prasad Government medical college, Kangra at Tanda Himachal Pradesh, India; 2 Department of Repertory, Homeopathic medical college, Chandigarh, India; 3 Department of ENT/oto laryngology Dr Rajendra Prasad Government medical college, Kangra at Tanda Himachal Pradesh, India; 4 Department of radiation Oncology, All India institute of medical sciences, Bilaspur, Himachal Pradesh, India

**Keywords:** Nuclear medicine, Awareness, Knowledge, Appropriate use, Interns, Residency trainees, Faculty

## Abstract

**Objective(s):**

Despite significant progress in the field of nuclear medicine, basic nuclear medicine awareness and understanding among clinicians remains unsatisfactory, leading to under utilization of nuclear medicine modalities. To evaluate the awareness and knowledge regarding nuclear medicine and appropriate use of Nuclear medicine modalities, among medical students and faculty members.

**Method:**

In this descriptive cross sectional study, a self timer limited objective questionnaire based on Google forms was distributed to the study population and scores obtained by the participants were analyzed.

**Results:**

Percent scores range for intern, residency trainees, and senior resident/faculty groups for general awareness were 16-46%, 37-58% and 62-91% and for knowledge and appropriate use were 7-21%, 28-43%, and 35-85% respectively. Overall, 61% of the participants had poor awareness and knowledge regarding nuclear medicine modalities. None of the participants had received nuclear medicine exposure or education during their academics or training. Only 49% of the participants considered utilizing nuclear medicine modalities for their patient management.

**Conclusion:**

Undergraduate interns and residency trainees had a poor to fair level of awareness and knowledge regarding nuclear medicine. Hence creating more awareness in early stages of their career by incorporating Nuclear medicine basic education in medical undergraduate curriculum is required. The senior residents/faculty members had a moderate to good level of awareness and knowledge but still improvement in their knowledge would lead to a more appropriate and better utilization of nuclear medicine modalities for optimum patient management in a variety of clinical settings.

## Introduction

 Nuclear medicine involves administration of trace amounts of radioactive material called radionuclides or radio tracers for the diagnosis, and management of various benign and malignant conditions (1, 2).These radionuclides or radiotracers emit gamma rays or positrons, which arethen used for imaging the patient’s body, with a gamma camera or a PET scanner. Some of the radionuclides used in nuclear medicine emit alpha or beta particles, and used for targeted radionuclide therapy (1-3). Nuclear medicine is often more sensitive andallows a more accurate and earlier diagnosis of underlying pathology because of its ability to detect changes in biological and physiological processes which usually precede the structural and morphological changes evident on anatomical imaging(4,5). 

 With development of PET and SPECT and their integration with anatomic modalities like CT and MRI, nuclear medicine has now evolved into a hybrid imaging modality, demonstrating much clinical value and benefit in routine and standard medical care (6). Diagnostic Nuclear medicine utilizes gamma camera SPECT for functional thyroid and parathyroid scans, bone scans for bone pathologies and skeletal metastasis, DTPA and DMSA scans for renalfunction and cortical pathologies and cardiac scans for CAD and myocardial viability. Hybrid PET/CT imaging using ^18^F-FDG is being increasingly utilised worldwide for diagnosis, staging, restaging and recurrence detection in oncology patients. And also for diagnosis and for assessment of disease activity and treatment response in various infective and inflammatory conditions (7, 8). 

 Besides ^18^F-FDG, other tracers such as ^68^Ga-DOTANOC, ^68^Ga-PSMA,^18^F-DOPA and many others are also being used for diagnosis and localization of a variety of tumours, infective, vascular and neurodegenerative disorders (9).

 It is therefore required that medical students in training, as well as practicing clinicians/ consultants, have a basic knowledge and appropriate understanding of clinical indications and contraindications of nuclear medicine modalities.

 Despite the progress in field of nuclear medicine, studies undertaken in some countries, have reported a variable and inadequate nuclear medicine teaching and education and a unsatisfactory level of awareness and knowledge regarding appropriate use of nuclear medicine modalities among medical students junior doctors and clinicians (10, 11). However, to the best of our knowledge, no such studies have been carried out in Indian context, and more so in northern region of the country.

 Therefore, we undertook this study with an aim to assess undergraduate medical students as well as post graduate residents, senior residents and faculty members, from two academic medical colleges in our state, with regards to their awareness towards nuclear medicine as a speciality and appropriate utilization of nuclear medicine modalities for patient management.

## Methods

 In this descriptive cross sectional study, a questionnaire (Annexure 1-3) based on a timer limited Google form was distributed to participants from two of the largest and oldest academic medical institutes (Indira Gandhi medical college, Shimla and Dr Rajendra Prasad Government medical college, Kangra at Tanda) in Himachal Pradesh, India.

 All participation was completely voluntary. Each participant was encouraged to read the introduction part of the survey form which outlines the objectives of the study and gives assurance that all data obtained from the study is anonymous. The project was approved by the institute ethics committee.

 The questionnaire was prepared by an experienced nuclear medicine specialist; validity of questions and their correct responses was confirmed by another nuclear medicine specialist and by consensus of standard nuclear medicine reference text books.

 Questionnaire consisted of three parts: A) 6 Multi choice questions on general awareness regarding nuclear medicine( annexure1), B) 14 single best choice questions on appropriate use and contraindications for nuclear medicine modalities ( annexure2), and C) 6 non scoring questions regarding their previous nuclear medicine education and clinical practices ( annexure 3).

 To minimize any online help while answering the questions, participants were asked to submit the responses within allotted time frame of 25 minutes. An auto self-close timer was integrated to the Google form to limit submission time to 25 minutes once survey was opened with help of an add on service from Quilgo.com. In addition, submission of online responses was allowed only once per participant and no modifications of responses allowed once submitted.

 Minimum score was 0 and maximum score was 2 for each scoring question. For question with more than one correct answer, split/fractional scoring was used and added up for each correct response to generate cumulative score for that question. Maximum cumulative possible score for the awareness part was 12 whereas maximum cumulative score for knowledge part of questionnaire was 28. Overall percentage scores for the respondents for both parts of the questionnaire were then calculated. A total score of less than 50% was rated as poor, 50-75% was moderate and >75% was considered as good.

***Statistical analysis***

 Data analysis was carried out using SAS (Version 9.4 for Windows). Descriptive statistics were applied to the data. Categorical data were presented as frequencies and percentages, while continuous data were presented using mean, and standard deviations. Significance of difference in the means scores for awareness and knowledge levels among the groups were compared with one way ANOVA and post-hoc Tukey's HSD (honestly significant difference) test. P-values <0.05 indicate significant results.

***Observations and analysis***

 A total of 346 participants from 2 academic institutes enrolled for survey and answered the questionnaire. Of these, 32 participants could not complete the survey within allocated time frame, and hence were excluded from the study. So the final study included 314 respondents, of which 142(~45%) were undergraduate interns, 68(~22%) postgraduate residency trainees (PG 1^st^, 2^nd^ and 3^rd^ year residents) and 104(~33%) were senior residents/faculty members.

 Means scores for interns, residency trainees and senior residents/faculty groups for awareness questionnaire were 0.48, 1.18 and 1.48 while mean scores for knowledge and appropriate use of nuclear modalities questionnaire were 0.21, 0.38 and 0.92 respectively (table1 and 2).

**Table1:** Mean scores of the three respondent’s categories regarding awareness towards Nuclear medicine and nuclear medicine modalities

**Category**	**Interns Group (n=142)**		**Residency Trainees (n=68)**		**Senior residents and faculty (n=104)**	
**Question**	**Mean score**	**S.D**	**Mean score**	**S.D**	**Mean Score**	**S.D**
**Q.1**	0.7		1.5		1.8	
**Q.2**	0.5		1.1		1.6	
**Q.3**	0.5		1.0		1.2	
**Q.4**	0.5		1.2		1.4	
**Q.5**	0.4		1.1		1.4	
**Q.6**	0.3		1.2		1.5	
**Mean**	0.48	0.13	1.18	0.17	1.48	0.20

**Table2:** Mean scores of the three respondent’s categories regarding Knowledge and appropriate use of nuclear medicine modalities

**Category**	**Interns Group (n=142)**		**Residency Trainees (n=68)**		**Senior residents and faculty (n=104)**	
**Question**	**Mean score**	**S.D**	**Mean score**	**S.D**	**Mean Score**	**S.D**
**Q.1**	0.25		0.30		1.1	
**Q.2**	0.20		0.40		1.0	
**Q.3**	0.22		0.31		0.9	
**Q.4**	0.30		0.44		1.1	
**Q.5**	0.17		0.43		0.88	
**Q.6**	0.25		0.50		1.0	
**Q.7**	0.14		0.37		0.80	
**Q.8**	0.17		0.33		0.92	
**Q.9**	0.40		0.64		1.2	
**Q.10**	0.22		0.35		1.1	
**Q.11**	0.19		0.30		0.7	
**Q.12**	0.17		0.37		0.61	
**Q.13**	0.22		0.35		0.92	
**Q.14**	0.14		0.34		0.84	
**Mean**	0.21	0.06	0.38	0.1	0.92	0.16

 Percent score ranges for interns, residency trainees and senior residents/faculty groups for awareness were 16-46%, 37-58% and 62-91% while percent scores for knowledge and appropriate use questionnaire were in range of 7-21%, 28-43%, and 35-85% respectively. 

 Senior resident /faculty group had the highest number of participants with correct individual responses while interns had the lowest number of correct responses to the questionnaire (table 3 and 4).

**Figure1:**
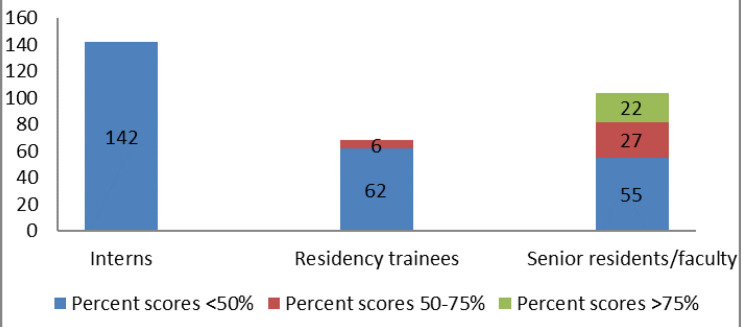
Number of respondents in each of three categories with overall percent scores to questions regarding awareness, Knowledge and appropriate use of nuclear medicine modalities

 Overall, 259(~82%) participants scored less 50%, 33(~11%) scores in 50-75% range while only 22(~7%) scored more than 75% (Figure1).

**Table3:** Correct response distribution of three respondent’s categories regarding knowledge and appropriate use of nuclear medicine modalities

**Category**	**Interns Group (n=142)**		**Residency Trainees (n=68)**		**Senior residents and faculty (n=104)**	
**Question**	**Correct** **response (n)**	**Percentage** **of Correct responses**	**Correct response (n)**	**Percentage** **of Correct responses**	**Correct response( n )**	**Percentage** **of Correct responses**
**Q.1**	18	12%	10	14%	58	55%
**Q.2**	14	10%	24	35%	62	59%
**Q.3**	16	11%	14	20%	50	48%
**Q.4**	22	15%	22	32%	60	57%
**Q.5**	12	8%	18	26%	46	44%
**Q.6**	18	12%	24	35%	54	52%
**Q.7**	10	7%	16	23%	42	40%
**Q.8**	12	8%	18	26%	48	46%
**Q.9**	30	21%	32	47%	66	63%
**Q.10**	16	11%	12	17%	70	67%
**Q.11**	14	10%	10	14%	36	34%
**Q.12**	12	8%	16	23%	32	30%
**Q.13**	16	11%	22	32%	48	46%
**Q.14**	10	7%	14	20%	44	42%

**Table4:** Minimal maximal and percent scores (brackets) of the three respondents categories to awareness and Knowledge regarding nuclear medicine modalities

**Category**		**Interns**	**Residency Trainees**	**Senior residents and faculty**
**Awareness score** **( Total score 12)**	Minimal score (% score)	2 (16%)	4.5 (37%)	7.5 (62%)
	Maximum score (% score )	5.5 (46%)	7 (58%)	11 (91%)
**Knowledge score** **( Total score 28)**	Minimal score (% score )	2 (7%)	8 (28%)	10 (35%)
	Maximum score (% score)	6 (21%)	12 (43%)	24 (85%)

 Of the 104 senior resident and faculty group respondents, 47(~45%) had completed their post-graduation or senior residency from central institutes, i.e., Post Graduate Institute of Medical Education and Research, Chandigarh and All India Institute of Medical Sciences New Delhi while others n=57(-~55%) had been trained in state medical colleges Dr RPGMC and, and IGMC Shimla,). Percent scores for former group ranged between 85 to 91% for awareness and ~70 to 85% for knowledge and appropriate use of nuclear medicine modalities respectively. For latter group, the percent scores were in range of ~62 to 65% and ~35 to 50% respectively. Mean scores for awareness questionnaire of senior residents/ faculty groups trained from PGIMER/AIIMS was 1.7 while mean scores of group trained from state medical colleges was 1.18. Similarly mean scores for knowledge and appropriate use of nuclear modalities questionnaire for the two groups were 1.2 and 0.7 respectively.

***Responses to questions regarding awareness towards nuclear medicine***

 Of the 314 respondents, 149(~46%) of the respondents answered that that they were aware about nuclear medicine as an independent medical speciality.

 142(45%) respondents answered that one can join MD/DNB nuclear medicine after completing MBBS, while others misinterpreted NM as super speciality that could be joined after MD in medicine or radiology.

 Only 86(~27%) of the respondents answered correctly that nuclear medicine scans involve radiation exposure.

 116(~37%) respondents correctly identified FDG PET scan, thyroid scan and DMSA/DTPA scans as nuclear medicine modalities, while 168 respondents wrongly answered DEXA scan as a nuclear medicine modality. 

 113(36%) of the respondents correctly answered that nuclear medicine deals with diagnostic scans and therapeutic procedures using radioactive molecules.

 Only 98(31%) of the respondents correctly answered that nuclear medicine modalities are used for both oncological and non-oncological conditions of varied etiologies while 124( 39%) respondents answered nuclear medicine is used for oncology patients only.

***Responses to questions regarding knowledge and appropriate use of nuclear medicine modalities***

 Of the 314 respondents, only 86(~27%) of the respondents answered correctly that nuclear medicine scans involve radiation exposure.

 Only 100(~32%) of the respondents answered correctly that nuclear medicine scans allow early detection, staging of the disease and can detect disease recurrence.

 128(~40%) of the respondents answered correctly that radioiodine therapy is used to treat Thyrotoxicosis/hyperthyroidism due to Graves’ disease/autonomous nodules.

 Only 104(~33%) of the respondents answered correctly that EC/DTPA can be used to assess the percent relative function and drainage pattern of kidneys.

 Only 78(25%) of the respondents answered correctly that Cardiac/Myocardial perfusion scan is the non-invasive modality can be most helpful in diagnosis, risk stratification and evaluation in a patient with ACS/ or suspected coronary artery disease.

 Only 63(~20%) of the respondents answered correctly that FDG PET/CT can be used to monitor disease activity and treatment response in condition like TB, vasculitis and sarcoidosis.

 Only 76(~24%) of the respondents answered correctly that FDG PET/CT can be used to detect an underlying pathology in a patient with fever of unknown origin.

 Only 86(~27%) of the respondents answered correctly that Pregnancy should be ruled out before subjecting the patient to nuclear medicine procedures.

 Only 68(~21%) of the respondents answered correctly that Pregnancy is an absolute contraindication to radionuclide/nuclear medicine therapy.

***Exposure and attitude towards nuclear medicine***

 Regarding exposure to nuclear medicine modalities and basic orientation, 267(~85%) of the respondents admitted that they had no exposure or basic orientation courses with respect to nuclear medicine during their academic training. 

 More than 95% participants also felt that they would like to have basic orientation sessions for understanding of nuclear medicine modalities in order to prescribe them efficiently more patient management in their OPD and wards.

 Among those involved directly in patient care and management decisions, i.e, residency trainees, SR( senior resident), and faculty 84/172(~49%) participants answered in affirmative with regard to their consideration for referral and utilization of nuclear medicine modalities for their patient management during their OPDs and ward rounds.

 More than 90% of the participants considered that a fully functional and equipped nuclear medicine department in their hospital will be helpful in improving the patient management care.

## Discussion

 The results from our study show an unsatisfactory level of awareness and understanding of nuclear medicine awareness and knowledge and understanding among junior doctors and senior residents and faculty.

 Overall, 82% of respondents scored in the “poor” category, 11% of respondents scored in the “moderate” category, and only 7% of respondents scored in the “good awareness and knowledge” category. Whereas, in study by Dhoodat (10), 43% of respondents scored in the medium (51-75%) group. This difference in results could be explained by the fact that the respondents in that study had received short postings in nuclear medicine departments while majority of respondents in our study had no formal nuclear medicine exposure or postings. In another study by Zakavi et al (12), 62% general physicians were reported to have a poor knowledge regarding applications of nuclear medicine.

 Looking at category or group wise data, percent score range for interns, residency trainees and senior residents/faculty groups were 16-46%, 37-58% and 62-91% respectively for nuclear medicine awareness questionnaire whereas the percent scores for three groups were in the range from 7-21%, 28-43%, and 35-85% respectively for appropriate use of nuclear medicine modalities. This shows that junior doctors had a poor level of awareness and knowledge regarding nuclear medicine. Similarly in a study Dasgupta (13) showed poor awareness and knowledge among junior doctors. However, senior residents/ faculty were not assessed in that study.

 In the present study, there were significant differences in mean scores between interns, residency trainees and senior resident/faculty groups for both general awareness (P alue<0.00001;

F-statistic: 53.52 and F critical value: 3.68) and knowledge regarding appropriate use of nuclear medicine modalities (P value<0.00001; F-statistic: 137.5 and F critical value: 3.2).

 There was also significant differences in mean scores for nuclear medicine awareness between the two groups of senior resident/faculty groups who had been trained at PGIMER/ AIIMS and in state medical colleges (P value~0.00004; F-statistic: 47.5 and F critical value: 4.9) and knowledge regarding appropriate use of nuclear medicine modalities (P value< 0.000003; F-statistic: 94 and F critical value: 4.2) (table 5 and table 6).

**Table 5:** Comparison of mean scores of the senior resident and faculty categories regarding awareness towards nuclear medicine

**Category**	**Senior residents and faculty trained ** **at PGIMER /AIIMS (n=47)**		**Senior residents and faculty trained in state medical colleges (n=57)**	
**Question**	**Mean score**	**S.D**	**Mean Score**	**S.D**
**Q.1**	1.8		1.5	
**Q.2**	1.7		1.1	
**Q.3**	1.7		1.0	
**Q.4**	1.6		1.2	
**Q.5**	1.7		1.1	
**Q.6**	1.7		1.2	
**Mean**	1.7	0.06	1.18	0.17

**Table 6:** Comparison of mean scores of the senior resident and faculty categories regarding Knowledge and appropriate use of nuclear medicine modalities

**Category**	**Senior residents and faculty trained ** **at PGIMER /AIIMS (n=47)**		**Senior residents and faculty trained in state medical colleges (n=57)**	
**Question**	**Mean score**	**S.D**	**Mean Score**	**S.D**
**Q.1**	1.1		0.6	
**Q.2**	1.2		0.7	
**Q.3**	1.0		0.5	
**Q.4**	1.2		0.9	
**Q.5**	1.0		0.7	
**Q.6**	1.3		0.9	
**Q.7**	1.1		0.6	
**Q.8**	1.2		0.5	
**Q.9**	1.7		1.3	
**Q.10**	1.6		0.7	
**Q.11**	1.2		0.5	
**Q.12**	0.9		0.5	
**Q.13**	0.8		0.4	
**Q.14**	1.1		0.6	
**Mean**	1.2	0.25	0.7	0.24

 Approximately 85% of respondents reported that they did not have any nuclear medicine education or basic awareness training during their under graduate or post graduate training and more than 90% participants answered that they would like to have basic orientation sessions in nuclear medicine, which is similar to findings of study by Dhoodat (10) where majority of respondents (87%) agreed that their NM education was insufficient during undergraduate training and 95% expressed interest in getting more information about NM to aid in referring patients for NM scans. In another a study by Adambounou K et al (14), 87.32% of the respondents felt it was important to spend a semester in a nuclear medicine department during their training.

 None of the participants graduated or post graduated from the two state medical colleges (n=267;~85%) have had any exposure to nuclear medicine postings or even basic orientation sessions with nuclear medicine departments, during their undergraduate training or post graduate residency, as the nuclear medicine department was ill equipped in one institute and only recently created in the other institute with no nuclear medicine infra structure at the time of this survey, which explains their low levels of nuclear medicine literacy. Similarly Adambounou K et al (14) also explained low rate of nuclear medicine awareness as a result poor accessibility of nuclear medicine services in French-speaking Africa where 37.46% of radiologists had no nuclear medicine department in their country and only 27.46% had one in their city of practice.

 In our study, on an average only ~30% of the respondents correctly knew indications for appropriately utilizing nuclear medicine modalities for optimizing patient care and management, with maximum respondents demonstrating better awareness and knowledge for utilizing nuclear medicine modalities in fields of endocrinology (40%) and urology (33%). Whereas in another study by Adambounou et al (14) in subSaharan Africa, the average number of respondents who knew the clinical indications for the main fields of nuclear medicine was (56.8%) with extremums of 66 (46.5%) in nuclear neurology and 98 (69%) in nuclear endocrinology. However in that study the respondents were radiologists, some of whom had internship or worked in nuclear medicine departments. Results from our study indicate that a lack of appropriate knowledge explains why only 49% of the participants involved directly in patient management decisions in OPD (outpatient department) and wards, considered utilizing nuclear medicine modalities in management of their patients, which is quite low, despite the benefits nuclear medicine has to offer in terms of earlier detection and higher sensitivity for management of a large number of pathological conditions (1-5).

 In contrast, senior resident and faculty members category had significantly better awareness and knowledge compared to their juniors. This is on the expected lines as some of the senior residents and faculty members have had their training in institutes likes PGIMER Chandigarh and AIIMS New Delhi, where unlike state institutes, nuclear medicine is an essential part of patient management and academic activities and discussions, and hence this explains their higher level of awareness and knowledge regarding the same. Moreover, senior residents and faculty members do get some exposure to nuclear medicine modalities and their utilization via participation in various seminars and conferences and journal articles during their academic and clinical at different levels and institutes.

 Nuclear medicine departments in the two state medical colleges surveyed in the present study are ill equipped and only partially functional. Considering this fact, a response was also sought from the participants on need make NM departments fully equipped. More than 90% of the participants answered that a fully functional and equipped nuclear medicine department in their hospital is essential and will be helpful in improving the patient care and management. This response is similar to response achieved from participants in a study by Adambounou Kokou et al (14) where 95.77% of the respondents considered creation of a nuclear medicine service in their respective countries to be essential.

 Radiology and nuclear medicine in many countries including India are still considered an adjunct or ancillary subject to clinical specialities and not incorporated and implemented in undergraduate medical school curricula thus leading to a low level of nuclear medicine awareness and knowledge in graduating medical students (15-17). A few studies undertaken in European and African countries have also revealed high level of variation and differences in the undergraduate teaching of nuclear medicine curriculum content and teaching methods (12, 16, 17). Such inadequate learning or lack of training can have a detrimental effect on patient management as the majority of medical graduates and practicing clinicians have little or limited knowledge of various aspects of nuclear medicine modalities and their appropriate use (24). This results in inadequate and in appropriate referral (18, 19) poor awareness of appropriate indications, contraindications and radiation risks involved (18-21).

 Therefore a short rotatory posting in nuclear medicine department, or cased based classes or lectures in nuclear medicine with emphasis on education and appropriateness should be incorporated into curriculum of the under-graduate students in final clinical year or internship period. Such an exposure can significantly improve their awareness and knowledge (13, 22, 23), and enable them to consider an appropriate nuclear medicine modality more often as part of the management of their patients in clinical practice and their future careers (13). Furthermore, nuclear medicine workshops or inter departmental presentations can allow clinicians directly involved in patient care, such as senior residents and faculty members, in improving their understanding of the appropriate indications, benefits, contraindications and also the costs involved in justified and optimum utilization of these modalities in improving patient care and outcomes (24). 

### Limitations of the study

 This study was limited to two academic institutes of one state only. The overall response rate was approximately 54%. Since not all undergraduate students, residency trainees and senior residents/faculty members enrolled or participated in the survey therefore generated data from the sample may not be exactly representative of the entire study population. 

 Therefore in future study including other academic institutes with a larger sample population would further reflect the levels of awareness and knowledge regarding nuclear medicine among junior and senior doctors in medical institutes in North India. 

## Conclusion

 Current level of awareness and knowledge of undergraduate interns in the field of nuclear medicine is quite unsatisfactory. Comparatively, senior residents and faculty members have better awareness, but still there are obvious gaps in their knowledge about appropriate indications and optimum use of nuclear medicine modalities in routine patient management. There is a definite need to incorporate a short nuclear medicine posting or basic nuclear medicine education in the undergraduate medical curriculum. Further, interactive inter-departmental lectures, workshops or CMEs would be beneficial in addressing these lacunae and thus improve utilization of nuclear medicine modalities for optimising the patient care and proper utilization of nuclear medicine modalities.

## Abbreviations used

 PGIMER: Post Graduate Institute of Medical Education and Research

AIIMS: All India Institute of Medical Sciences

JR: Junior Resident

SR: Senior Resident

OPD: Out Patient Department 

## Author Declarations

 We confirm that this manuscript has not been published in your journal or elsewhere and is not under consideration by another journal.

1. All authors have contributed significantly, approved content of the manuscript and agree with its submission to Asia Oceania Journal of Nuclear Medicine & Biology.

2. All author declare that there is no financial support or relationships that may pose conflict of interest.

## Conflicts of interest

 No conflicts of interest to report.

## Data availability statement

 All relevant data has been preserved and is reproducible.

## Ethics statement

 This cross sectional study was based on a survey among undergraduate interns, residency trainees and senior residents and faculty members from two academic medical institutes in Himachal Pradesh India. Prior ethical approval from institute ethical committee was obtained for the survey.

## Participant consent

 Participants were informed about intent of the survey and data collection process, that their participation was entirely voluntary, and that their identity would be kept anonymous.

## Funding

 No funding sources to report.
